# The Physiological Bases of Hidden Noise-Induced Hearing Loss: Protocol for a Functional Neuroimaging Study

**DOI:** 10.2196/resprot.9095

**Published:** 2018-03-09

**Authors:** Rebecca Susan Dewey, Deborah A Hall, Hannah Guest, Garreth Prendergast, Christopher J Plack, Susan T Francis

**Affiliations:** ^1^ Sir Peter Mansfield Imaging Centre School of Physics and Astronomy University of Nottingham Nottingham United Kingdom; ^2^ National Institute for Health Research Nottingham Biomedical Research Centre Nottingham United Kingdom; ^3^ Otology and Hearing Group, Division of Clinical Neuroscience School of Medicine University of Nottingham Nottingham United Kingdom; ^4^ Manchester Centre for Audiology and Deafness University of Manchester Manchester United Kingdom; ^5^ National Institute for Health Research Manchester Biomedical Research Centre Manchester Academic Health Science Centre, Central Manchester University Hospitals NHS Foundation Trust Manchester United Kingdom; ^6^ Department of Psychology Lancaster University Lancaster United Kingdom

**Keywords:** functional magnetic resonance imaging, auditory pathways, auditory brain stem response

## Abstract

**Background:**

Rodent studies indicate that noise exposure can cause permanent damage to synapses between inner hair cells and high-threshold auditory nerve fibers, without permanently altering threshold sensitivity. These demonstrations of what is commonly known as hidden hearing loss have been confirmed in several rodent species, but the implications for human hearing are unclear.

**Objective:**

Our Medical Research Council–funded program aims to address this unanswered question, by investigating functional consequences of the damage to the human peripheral and central auditory nervous system that results from cumulative lifetime noise exposure. Behavioral and neuroimaging techniques are being used in a series of parallel studies aimed at detecting hidden hearing loss in humans. The planned neuroimaging study aims to (1) identify central auditory biomarkers associated with hidden hearing loss; (2) investigate whether there are any additive contributions from tinnitus or diminished sound tolerance, which are often comorbid with hearing problems; and (3) explore the relation between subcortical functional magnetic resonance imaging (fMRI) measures and the auditory brainstem response (ABR).

**Methods:**

Individuals aged 25 to 40 years with pure tone hearing thresholds ≤20 dB hearing level over the range 500 Hz to 8 kHz and no contraindications for MRI or signs of ear disease will be recruited into the study. Lifetime noise exposure will be estimated using an in-depth structured interview. Auditory responses throughout the central auditory system will be recorded using ABR and fMRI. Analyses will focus predominantly on correlations between lifetime noise exposure and auditory response characteristics.

**Results:**

This paper reports the study protocol. The funding was awarded in July 2013. Enrollment for the study described in this protocol commenced in February 2017 and was completed in December 2017. Results are expected in 2018.

**Conclusions:**

This challenging and comprehensive study will have the potential to impact diagnostic procedures for hidden hearing loss, enabling early identification of noise-induced auditory damage via the detection of changes in central auditory processing. Consequently, this will generate the opportunity to give personalized advice regarding provision of ear defense and monitoring of further damage, thus reducing the incidence of noise-induced hearing loss.

## Introduction

### Background and Rationale

Noise exposure is the main cause of preventable hearing loss worldwide, as identified by the World Health Organization [1]. Noise exposure can occur environmentally [2], occupationally in the workplace [3], and recreationally during personal leisure time [4]. Damage from noise exposure can manifest at many points along the auditory pathway, including the sensory hair cells in the cochlea, and the connections between hair cells and nerve cells (synaptopathy, [5]). Damage to the auditory nerve can also lead to tinnitus (perception of sound in the absence of external sound) and hyperacusis (diminished tolerance of moderate- to high-level sounds) [6].

Controlled experiments on the effects of noise exposure on the cochlea use an animal model of acute noise trauma. A striking discovery showed that noise exposure can cause substantial neural damage without a reduction in threshold sensitivity. Mice exposed to a 100 decibel sound pressure level (dB SPL) stimulus for just 2 hours permanently lost up to half of their hair-cell or auditory-nerve synapses in certain frequency regions (cochlear synaptopathy), despite a complete recovery of thresholds for sounds in quiet [7]. Several weeks after exposure, auditory-nerve activity (as measured by electrophysiological auditory evoked potentials; AEPs) was normal at low sound levels but reduced at suprathreshold levels. This suggests that the damage affects auditory nerve fibers with high thresholds, which are also thought to be the fibers that encode acoustic information at medium to high levels and in background noise [8]. These findings have been replicated in the guinea pig [9] and chinchilla [10], suggesting a general mammalian effect. These studies suggest that even moderate noise exposure can cause substantial damage to the auditory nerve, while leaving hair cells macroscopically intact. Particularly troubling is that neuropathy has also been reported in mice exposed to a stimulus of just 84 dB SPL (a level of noise exposure that is below the Health and Safety Executive action point for health surveillance) for 168 hours per 1 week [3,11]. However, confidence in this finding is reduced by the observation that synaptic counts in the exposed mice were similar to those of control mice in previous studies [12]. Nevertheless, the prediction from these acute noise trauma models is that human noise exposures, accumulated over a lifetime, exert a similar causative effect by damaging the synapses between the inner hair cells and the auditory nerve fibers leading to nerve fiber degeneration.

Hearing ability is typically assessed using pure tone audiometry, which measures the ability to detect quiet sounds by determining the threshold for single-frequency tones up to 8 kHz [13]. Until recently, it had been assumed that hearing loss results mainly from damage to the sensory hair cells in the cochlea [14]. However, the literature cited here suggests that primary damage to neural structures may precede hair cell loss and may not be detectable by pure tone audiometry. Hence, cochlear synaptopathy with perceptual effects is sometimes referred to as hidden hearing loss [15].

The auditory brainstem response (ABR) is the AEP of the brainstem and vestibulocochlear nerve. The amplitude of each peak of the ABR reflects the number and synchronicity of neurons firing, and the latencies represent the speed of transmission of the AEP [16]. The amplitudes of waves I and V of the ABR, and often the wave I/V amplitude ratio are reported, and either a reduction in the amplitude or the ratio between these amplitudes has been related to tinnitus [16,17]. Conversely, other studies have failed to replicate these findings [18,19].

Noise exposure leads to sensorineural damage, which degrades the information carried by the nerve from the ear to the brain [14]. Studies [20,21] suggest that people with a history of noise exposure, but with normal pure tone audiometric thresholds, experience problems with sound discrimination, particularly understanding speech in noisy environments. For example, noise-exposed workers demonstrated worse speech recognition in the presence of multitalker babble at −5 dB signal-to-noise ratio compared with controls [20], and high-risk college students scored lower on word recognition in noise than did their low-risk counterparts [21]. However, contradictory to this, some studies find no evidence of any link between noise exposure and speech perception deficits [22-24]. In addition to the immediate perceptual deficits that may result from damage to auditory nerve fibers and/or hair cells, it is known that noise damage earlier in life exacerbates hearing problems associated with old age [25]. From this, it is possible to infer that cumulative lifetime noise exposure may be predictive of hidden hearing loss due to the effect of exposure on hair cell or auditory nerve fiber aging. Furthermore, cumulative lifetime noise exposure may be predictive of tinnitus [19] and/or reduced sound-level tolerance.

The link between the physiological results and the perceptual deficits is as yet unclear, although some studies have small, but significant, associations between synaptopathy and deficits in auditory perception [26]. To date, however, we have found no discernible relation between any property of the electrophysiological ABR or frequency following response and either (1) noise exposure or (2) tinnitus in young adult humans. This has been determined from electrophysiological measures and lifetime noise exposure reports in (1) a group of 126 participants (aged 18-36 years) with matched audiometric thresholds up to 8 kHz [18] and (2) a group of 20 participants exhibiting tinnitus, when compared with controls matched for age and audiometry up to 14 kHz [19]. It is important to consider the literature when planning study design, and our protocol specifically addresses the following aspects. First, the study will collect cumulative lifetime measures of noise exposure, as opposed to recent short-term noise exposure measures [27]. Second, the study will recruit a large sample of 90 individuals spanning a range of ages. Third, care will be taken to ensure closely matched audiometric thresholds over the range of 500 Hz to 8 kHz, in contrast to the previous studies [17], and to assess high-frequency audiometric thresholds at 12 and 16 kHz which may influence ABR amplitudes [16,19]. The key extension compared with studies reported to date [18,19] includes assessment of functional magnetic resonance imaging (fMRI) measures from the brainstem, analogous to electrophysiological brainstem measures described above, and to perform this in a slightly older cohort aged 25 to 40 years, while still maintaining control on audiometric matching. From this study, we hope to achieve greater sensitivity to detect a relation between lifetime noise exposure and neurophysiology.

Previous fMRI studies have shown increased responses to auditory stimuli in the ascending auditory pathway and auditory cortex of individuals perceiving tinnitus and reduced sound-level tolerance [28-30]. This can be taken as evidence that physiological correlates of tinnitus perception and sound-level tolerance can be detected using fMRI. Additionally, this provides evidence for an association between central gain in the ascending auditory pathway and tinnitus or reduced sound-level tolerance. The overall aim of our 5-year research program is to understand the damage to the human auditory system that results from environmental noise, focusing on hidden hearing loss that is not detected by standard hearing tests. Our initial hypotheses are that noise exposure is associated with abnormal gain in the ascending auditory pathway and also with tinnitus, reduced sound-level tolerance, and impaired speech perception.

### Objectives

The primary objective of this neuroimaging experiment is to identify any central auditory biomarkers associated with hidden hearing loss. Specifically, we will determine whether fMRI techniques can detect physiological changes in the central auditory system of individuals with normal audiometric thresholds that are statistically associated with the degree of cumulative lifetime noise exposure. These changes are hypothesized to be detected in structures of the ascending auditory pathway, comprising the cochlear nucleus (CN), inferior colliculus (IC), medial geniculate body (MGB), and primary auditory cortex. We hypothesize that lifetime noise exposure will be associated with abnormal gain in the ascending auditory pathway, that is, increased fMRI response to auditory stimuli. To test this, we will first determine whether there are any differences between low and high noise exposure groups in the fMRI responses to broadband noise in the above key anatomically defined regions. Thereafter, we will assess whether there is any correlation between lifetime units of noise exposure and fMRI responses in the same regions.

A secondary objective is to investigate whether there are any additive contributions to these physiological changes attributable to tinnitus or diminished sound-level tolerance, conditions which are often comorbid with hearing problems.

A further secondary objective is to test the hypothesis that noise exposure is associated with a reduction of the ABR wave I and/or a reduction of the wave I/V amplitude ratio across low and high noise exposure groups, closely matched for audiometric thresholds.

Finally, this study will provide an opportunity to explore the relationship between ABR and fMRI measures in the ascending auditory pathway.

### Study Design

This study is designed to assess differences in individual fMRI responses in hypothesized, anatomically defined regions that may relate to units of lifetime noise exposure while controlling for age and audiometric threshold. Sound-related fMRI responses will be examined for differences that correlate with noise exposure using an analysis of variance (ANOVA). Presence of tinnitus and reduced sound-level tolerance will also be considered as factors of interest. Participants will be grouped in a factorial analysis, where the factors are noise exposure, tinnitus, and reduced sound-level tolerance.

The Organization for Human Brain Mapping Committee on Best Practice in Data Analysis and Sharing [31] states that reproducibility of fMRI studies can be improved by the process of preregistration [32]. Furthermore, there is a growing precedent for publishing fMRI protocols before completion [33]. In light of this, the methods here are reported in sufficient detail that they may be fully replicated and that any future publications resulting from this study can be cross-referenced to this paper.

## Methods

### Participants, Interventions, and Outcomes

#### Study Setting

The study protocol has been approved by the University of Nottingham School of Medicine Research Ethics Committee and will be conducted in accordance with these ethically approved procedures (reference: B/1207/2016). The study is part of a 5-year program that has been funded by the Medical Research Council (MRC) (grant number: MR/L003589/1 awarded to the University of Manchester). Progress is reported and monitored at annual Advisory Panel meetings attended by researchers at both universities, as well as a representative from the charity Action on Hearing Loss.

The magnetic resonance imaging (MRI) scanning will be conducted at the Sir Peter Mansfield Imaging Centre (SPMIC), a translational imaging center at the University of Nottingham. The data analysis will be conducted at the SPMIC and National Institute for Health Research Nottingham Biomedical Research Centre. All procedures will be performed by a member of research staff at the University of Nottingham. All study participants will give written informed consent.

#### Eligibility Criteria

Healthy adult volunteers aged 25 to 40 years will be included in the study. Participants will have clinically normal hearing thresholds as per BSA guidance on pure tone audiometry [13], that is, 20 dB hearing level or below from 500 Hz to 8 kHz. Exclusion criteria are contraindications for undergoing MRI, and signs of conductive hearing loss or ear disease identified by otoscopy and tympanometry [34]. Furthermore, any participants reporting exposure to explosions (large infantry weapons, light artillery or antiaircraft guns, large artillery weapons or naval guns, explosions) will be excluded from the study.

#### Participant Timeline

Table 1 shows a schematic diagram of the time schedule of enrollment and assessments for participants based on the Standard Protocol Items: Recommendations for Interventional Trials (SPIRIT) guidelines for reporting protocols of clinical trials [35].

#### Sample Size

In total, up to 90 individuals will be recruited into the study to one of two groups, depending on the noise exposure of the individuals recruited. Statistical power in fMRI research is influenced by 10 parameters, including study design and temporal autocorrelation [36,37], and we estimate that 30 individuals per group will provide acceptable reliability to differentiate between the groups with 80% power.

#### Recruitment

Recruitment will be stratified to ensure a balanced distribution of age and gender in each of the noise exposure groups. For example, we will aim for equal numbers of individuals reporting high and low noise exposure in the age ranges of 25 to 27, 28 to 30, 31 to 33, 34 to 36, and 37 to 40 years. Recruitment is expected to close in December 2017.

Participants will be recruited through advertisements displayed in public areas of University buildings (eg, library noticeboards, departmental noticeboards allocated to recruitment leaflets), on noticeboards in other public and private buildings (with the owners’ consent), Internet message boards, departmental websites, social media, local radio, and community magazines. We will specifically target buildings associated with activities that incur noise exposure, for example, music technology departments and live music venues.

Potential participants will be given an electronic copy of the information sheet, informed consent form, and MRI safety-screening questionnaire at least 24 hours before participating in the study to ensure that they have adequate opportunity to consider what is involved in the study. On arrival at the SPMIC, participants will be given paper copies of all study materials.

### Data Collection, Management, and Analysis

#### Screening Procedure

Suitability to undergo MRI will be determined by completion of a 19-item self-report screening questionnaire including questions about surgical history, implants and foreign bodies, epilepsy or blackouts, claustrophobia and tinnitus, tattoos, and willingness to remove all metal (eg, body-piercing jewelry, false teeth, hearing aid). Participants who do not meet the safety requirements and data-quality requirements for scanning will not be included in any part of the study.

The participant will undergo audiometry to determine hearing thresholds. Audiometry will be performed in a soundproof environment, free from distractions. Stimuli will be presented using an M-Audio M-Track Quad external sound card (M-Audio, Cumberland, Rhode Island, USA) over Sennheiser HDA300 audiometric headphones suitable for high-frequency audiometry (Sennheiser electronic GmbH & Co KG, Wedemark, Germany). Stimuli will be generated using in-house software written in Matlab (version 2016a, The MathWorks Inc., Natick, Massachusetts). Audiometry will be performed using a two-interval, two-alternative forced choice visually cued adaptive paradigm with a two-down one-up rule and a step size of 2 dB. The adaptive procedure will be stopped after 12 reversals, and the geometric mean of the signal level at the last eight reversals will be computed.

**Table 1:** Time schedule of enrollment and assessments for participants based on the SPIRIT guidelines. Questionnaires address biographical data, tinnitus and intrusiveness of tinnitus, hearing, and reduced sound-level tolerance.

Interaction		Email exchange	Study period	
		Pre-enrollment (timepoint *t*_−1_)	Enrollment (timepoint *t*_1_)	Assessment (timepoint *t*_2_)
**Enrollment**				
	Screen for age and MRI^a^ contraindications	X		
	Informed consent		X	
	Otoscopy and tympanometry		X	
	Audiometry		X	
**Assessments**				
	Questionnaires		X	
	Structured interview for lifetime noise exposure		X	
	ABR^b^		X	
	fMRI^c^			X

^a^MRI: magnetic resonance imaging.

^b^ABR: auditory brainstem response.

^c^fMRI: functional magnetic resonance imaging.

This paradigm will be used to establish monaural thresholds, in the left ear, followed by the right ear, at frequencies of 0.25, 0.5, 1.0, 2.0, 3.0, 4.0, 6.0, 8.0, 12.0, and 16.0 kHz. Stimuli used at frequencies 250 Hz to 8 kHz will be sinusoidal pure tones. Stimuli used at frequencies 12 and 16 kHz will be half-octave narrowband noise, to minimize the influence of ear canal resonances and threshold microstructure on measured thresholds.

#### Data Collection Methods: Lifetime Noise Exposure

Total noise exposure units will be estimated using a structured interview informed by the Noise Exposure and Rating Questionnaire [3]. Cumulative noise exposure over the lifetime will be assessed by a methodical and systematic approach, including noise exposure accrued in the settings of (1) occupational and educational, (2) social, and (3) gunshot and explosive noises.

For each setting, the participant will be asked to identify activities they engage in, in environments estimated to exceed 80 dB(A). For each activity, the participant will then be asked to estimate the level of exposure using a vocal effort scale comprising six different levels of vocal effort ranging from “raised voice” (87 dB(A)) to “shouting close to listener’s ear” (110 dB(A)). The participant will then be asked to estimate the duration for which they were in that environment/engaging in that activity, breaking this down into number of years, number of weeks per year, number of days per week, and number of hours per day. Finally, the participant will be asked to recall whether or not ear protection was used, what type of protection it was, and the proportion of time for which that ear protection was effective.

#### Data Collection Methods: Questionnaires

Participants will complete 3 questionnaires on (1) biographical data, including handedness, ethnicity, employment status and education; (2) tinnitus and hearing, including reduced sound-level tolerance, using the Tinnitus and Hearing Survey [38]; and (3) tinnitus intrusiveness, using the intrusiveness subscale of the Tinnitus Functional Index [39].

#### Data Collection Methods: Auditory Brainstem Response

Electrical activity will be recorded from all participants using the BioSemi ActiveTwo multichannel electroencephalography (EEG) system with active electrodes (BioSemi BV, Amsterdam, Netherlands). Three channels will be used; electrodes will be attached to the (1) vertex/Cz, (2) right mastoid, and (3) left mastoid with 10/20 electrode paste. Additional electrodes will be attached to the forehead, less than 3 inches apart, to form the ground (Common Mode Sense and Driven Right Leg).

Stimuli will be generated using in-house software written in Matlab and the same external sound card as for audiometry. Stimuli will be transmitted via shielded Etymotic ER3A transducers with disposable insert foam ear tips. ABR stimuli will consist of single-polarity high-pass filtered clicks (using a first-order Butterworth filter with high-pass cut-off=1.4 kHz) presented at 102 dB peak equivalent SPL. Click presentation will alternate between ears, at a rate of 22 s^−1^(11 s^−1^ per ear) for a total of 7000 clicks per ear. The recording will last approximately 10 min.

Recording will be performed in an electrically shielded, darkened, soundproof room. Participants will be lying flat or near-flat and covered with a blanket. Participants will be instructed to close their eyes, relax as much as possible, and told that they should feel free to fall asleep if they are able. Stimuli will be presented near-continuously throughout the relaxation and recording period. Recording will only commence when the EEG trace has stabilized and motion artifacts have subsided.

#### Data Collection Methods: Magnetic Resonance Imaging

fMRI will assess auditory responses from changes associated with cerebral blood flow, volume, and oxygenation using Blood-Oxygen-Level Dependent (BOLD) contrast. BOLD responses in hypothesized cortical and subcortical regions of interest (namely the primary auditory cortex and subcortical regions of CN, IC, and MGB) will be assessed on a subject-by-subject basis.

#### Scanning

**Figure 1:**
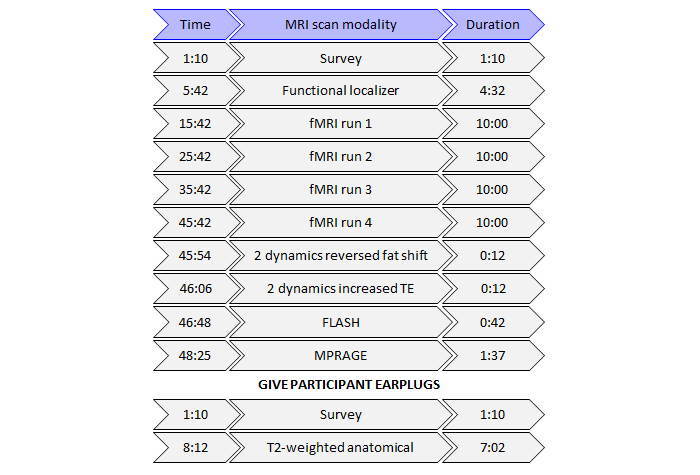
Schematic of the magnetic resonance imaging (MRI) protocol used in the study. fMRI: functional magnetic resonance imaging; TE: echo time; FLASH: fast low angle shot; MPRAGE: magnetization prepared rapid acquisition gradient echo.

All MRI measures for this study will be performed on a Philips 3.0 T Ingenia MR scanner (Philips Healthcare, Best, Netherlands) using a 32-element sensitivity encoding (SENSE) head coil. Subjects will wear noise-canceling headphones for the fMRI acquisition (see *Stimulus Presentation* below). A schematic of the MRI protocol is shown in Figure 1. Physiological data will be acquired throughout the scan session using respiratory bellows and a peripheral pulse unit for the purpose of performing RETROICOR (retrospective image-based correction; [40]) on the functional images to correct for physiological artifacts.

Functional MRI will be collected using a gradient echo (GE) echo-planar imaging (EPI) acquisition with high 1.5-mm isotropic spatial resolution and an echo time, TE, of 35 ms; flip angle of 90°; parallel imaging with SENSE factor of 2.5; field of view of 34.5 × 34.5 mm and a repetition time, TR, of 2 s. In total, 23 contiguous slices will be acquired with equidistant temporal slice spacing and descending slice scan order. Slices will be planned in a coronal oblique orientation to provide coverage of the brainstem and Heschl’s gyrus. Four fMRI runs will be collected in the scan session.

Before the main study fMRI runs, a functional localizer will be performed to confirm that the placement of the imaging slab includes the primary auditory areas. Responses will be elicited in these areas using a 10-Hz amplitude-modulated broadband noise stimulus of duration 24 s with a 40-s rest period, for a total of four repeats. To maximize statistical power of the functional localizer scan to detect activity in the primary auditory cortex, images will be acquired at a coarser spatial resolution of 2 mm isotropic and with a sparse repetition time, TR, of 8 s to ensure that auditory activation induced by the scanner noise has minimal influence [41].

**Figure 2:**
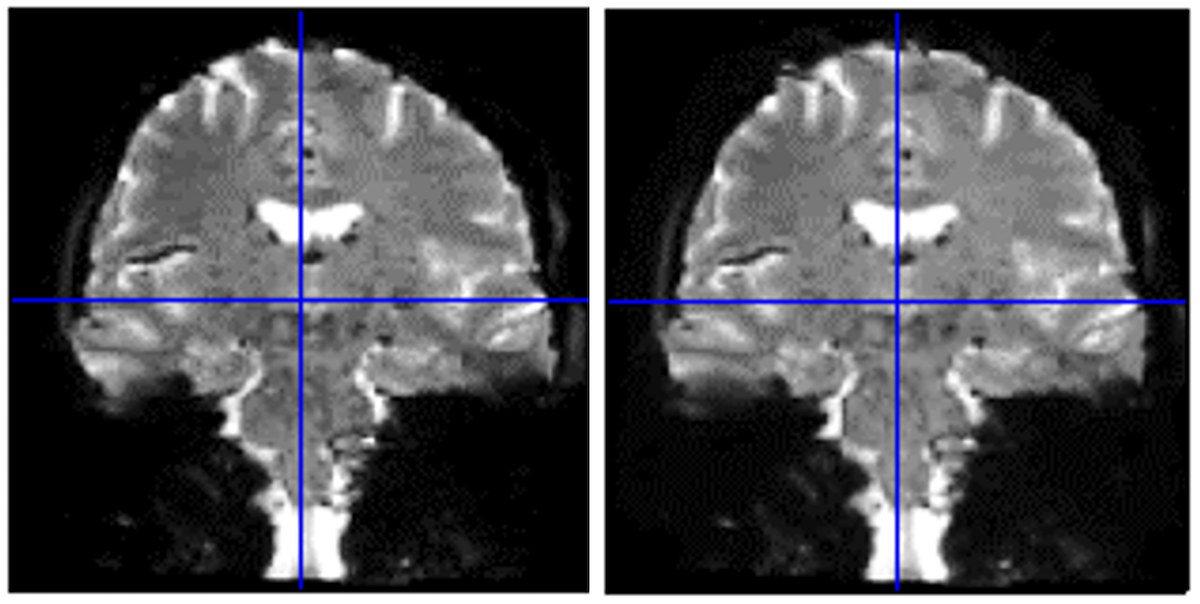
The effect of echo-planar imaging (EPI) distortion correction on the image. An uncorrected, distorted EPI (left) and corrected image (right).

Following the fMRI acquisition, additional images will be acquired for image distortion correction in the preprocessing stage, particularly important for studying group responses in the brain stem. This requires the acquisition of additional EPI image volumes with each of the following modifications: (1) reversal of the fat-shift direction, that is, right as opposed to left and (2) TE increase of 2 ms, that is, 37 ms as opposed to 35 ms. Figure 2 shows uncorrected EPI distortion side by side with an image that has been distortion corrected. To assist with linear coregistration of images between image types or contrasts, and for nonlinear coregistration to standard anatomical Montreal Neurological Institute (MNI) space (MNI, Template; Montreal Neurological Institute, Montreal, Canada), a distortion-free three-dimensional (3D) fast low angle shot (FLASH; [42]) image will be acquired with the same spatial resolution and geometry as the fMRI GE-EPI scans with a TE of 20 ms, TR of 880 ms, and flip angle of 18°. In addition, a whole-brain 3D anatomical magnetization prepared rapid acquisition gradient echo (MPRAGE) acquisition also with the same resolution and angulation as the GE-EPI data will be collected.

Following this session, the participants will be withdrawn from the MR scanner, and allowed to sit up and walk around if desired. They will then be given earplugs for insertion to ensure participant comfort before commencing a further scan to collect a high-resolution anatomical image. This will be a 3D T_2_-weighted turbo spin echo, TSE with TE of 278 ms, a TR of 2000 ms, and flip angle of 90°; with a field of view of 249 × 249 × 72 mm and reconstructed voxel size of 0.576 mm^3^.

#### Stimulus Presentation

The level of acoustic scanner noise during the high-resolution fMRI scans is reported by the scanner software to be 111.1 dB SPL. Auditory stimuli will be presented using the OptoActive Active Noise Cancellation Headphones system (Optoacoustics Ltd., Moshav Mazor, Israel). This provides MR-compatible delivery of high-quality sounds through circumaural headphones combined with 24-dB ear-defenders for passive attenuation of the scanner sound. Following an initial 16-s learning period, the active noise cancellation reduces the effective scanner sound to approximately 70 dB (accounting for both passive and active attenuation). Stimuli will consist of broadband noise, filtered (using a first-order Butterworth filter) between 1.4 and 4.1 kHz, and presented at 85 dB SPL.

Following an initial rest period (which includes the learning period for active noise cancellation), broadband noise will be presented for 24 s followed by a 42-s rest period. The task is passive listening and the stimulus will be presented for 8 repeats per run of 296 dynamics (10 min). A total of four 10-min fMRI runs will be performed, interspersed with a period of rest in which the researcher can communicate with the participant, and comfort can be ensured.

A fixation cross will be presented using a 32” BOLDscreen with a 1920x1080 widescreen LCD display (Cambridge Research Systems Ltd., Rochester, UK); subjects will view the screen using a mirror attached to the head coil placed approximately 10 cm from the face, will be instructed to maintain fixation on the cross throughout the functional scans, and will be reminded to do so at the beginning of each functional run.

#### Data Management

All data will be anonymized at source. All electronic data will be stored on a secure remote data storage drive maintained and backed up by the University of Nottingham.

### Data Preprocessing and Analysis

#### Lifetime Noise Exposure Data

**Figure 3:**
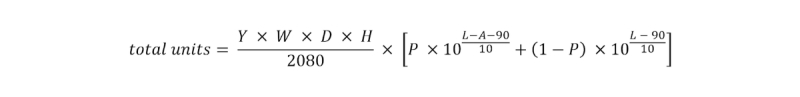
Calculation of total noise exposure units, where Y is the number of years of exposure, W the number of weeks per year of exposure, D the number of days per week of exposure, H the number of hours per day of exposure, L the level of exposure, in dB (A), as estimated by the participant, A the attenuation of hearing protective equipment (dB), and P the proportion of time protective equipment was worn, between 0 and 1.

Total noise exposure units will be calculated for each activity using the equation shown in Figure 3 [3], where *Y* is the number of years of exposure, *W* the number of weeks per year of exposure, *D* the number of days per week of exposure, *H* the number of hours per day of exposure, *L* the level of exposure, in dB (*A*), as estimated by the participant, *A* the attenuation of hearing protective equipment (dB), and *P* the proportion of time protective equipment was worn, between 0 and 1. Units for all activities will be calculated and then summed to give a participant’s total lifetime noise exposure, a measure linearly related to total energy of exposure above 80 dB(A).

#### Auditory Brainstem Response Data Preprocessing and Analysis

ABR data will be processed using software coded in Matlab, using a procedure informed by Guest et al [19]. For each ear, the time-course of the potential difference between Cz and the ipsilateral mastoid will be filtered (using a fourth-order Butterworth filter between 30 Hz and 1.5 kHz) and divided into epochs extending from 10 ms prestimulus to 13 ms post stimulus, after correcting for the 0.91 ms acoustic delay introduced by the tube connecting the transducer to the ear. Epochs with a root-mean-square amplitude of more than 2 standard deviations above the mean will be rejected. Data will then be averaged and the resulting waveform linearly detrended. An automatic peak-picking algorithm will then identify waves I and V of the ABR for each ear based on the time windows given in Table 2.

A secondary hypothesis focuses on differences in ABR wave I amplitude and in wave I/V amplitude ratio across low and high noise exposure groups. To answer this research question, individual ABR waveforms will be obtained for each ear separately. We will explore effects of the laterality of click presentation. To do this, we will perform a mixed ANOVA of derived measures (amplitude, latency) from each ear, with left and right ears as a within-subject factor and noise exposure as a between-subjects factor.

#### Magnetic Resonance Image Preprocessing

Image preprocessing will be performed using FSL version 5 brain-mapping software (Functional Magnetic Resonance Imaging of the Brain, FMRIB, Analysis Group, Oxford University, UK), SPM12 (Statistical Parametric Mapping version 12, Wellcome Trust Centre for Neuroimaging, University College London, UK), and in-house software toolboxes coded in Matlab. For individual participants, the fMRI timeseries will first undergo motion correction in SPM12. Data will then be distortion corrected using FSL’s TOPUP algorithm [43,44]. Data will then undergo physiological artifact correction for respiratory and cardiac effects using RETROICOR [40]. Following this, data will be spatially smoothed using a Gaussian kernel of full-width half-maximum 2 mm. Binarized masks of white matter and cerebrospinal fluid will be formed from the MPRAGE image using the segmentation tool in SPM12 and threshold at a level of 0.99999. These masks will be used to calculate mean time courses of white matter and cerebrospinal fluid (CSF) for use as nuisance covariates in the general linear model (GLM).

**Table 2:** Time windows used to constrain auditory brainstem response peak-picking algorithm.

ABR feature	Time window
Wave I peak	1.55-2.05 ms after stimulus peak
Wave I trough	0.3-1.0 ms after wave I peak
Wave V peak	5.1-6.6 ms after stimulus peak
Wave V trough	Baseline–see explanation in [19]

Individual subject data coregistration between the fMRI timeseries and the standard anatomical template will first be performed using the distortion-free MPRAGE/FLASH images, generating a matrix transform. This transform will then be applied to individual statistical parametric maps (SPMs) for region-of-interest (ROI) analyses in group or MNI space. This will allow the use of prespecified anatomically defined binary image masks of ROIs in the CN, IC, and MGB of the ascending auditory pathway, and additionally primary auditory cortex.

For interrogation of the sound-related activity at an individual level, ROIs can additionally be defined on high-resolution anatomical images. Coregistration between individuals’ anatomical scan and the fMRI timeseries will be performed using the distortion-free MPRAGE image, generating a matrix transform. This transform will then be applied to the hand-drawn ROI volumes for use on the fMRI timeseries. This may be preferable for analyses involving subcortical anatomical regions, as it will take into account the intersubject anatomical differences in the brainstem.

#### Functional Magnetic Resonance Imaging (fMRI) Data Analysis

Statistical analyses will be performed in SPM12 using a GLM which specifies the onset, offset, and duration of the auditory stimulus as predictor variables of interest, and the 6 motion-correction parameters and mean time courses of both white matter and CSF as nuisance covariates. Three predictor variables are required to optimally describe the shape of the fMRI response, which is known to change at different stages of the auditory pathway from a response that is sustained over the stimulus duration (eg, in the CN, IC) to one that is phasic with peaks just after stimulus onset and offset (eg, in the MGB, cortex) [45].

The fit of the individual fMRI timeseries to this GLM will be calculated and SPMs corresponding to the stimulus onset, offset, and duration will be generated for each participant. These SPMs contain information about the parameter estimates in the form of voxel-wise beta estimates for each predictor variable.

The primary objective is to identify any central auditory biomarkers associated with the estimate of cumulative lifetime noise exposure. This question will be addressed using 2 analysis strategies, each using a quantification of the sound-related fMRI responses in the predesignated ROIs. First, the individual SPM outputs will form the input to a second-level GLM that will account for intersubject variability across the sample. The model will again specify the onset, offset, and duration of the auditory stimulus as within-subject factors and with low- and high-risk noise exposure as a between-subject factor. This GLM will test the question of whether there are group differences in sound-related activity. The statistical significance of the findings generated by this model will be interpreted after applying a small volume correction using the group-level ROIs. Second, the individual SPMs will be interrogated to quantify the average parameter estimate (beta value) within the individual-level ROIs, separately for the 3 predictor variables of interest. Simple linear regression analyses will be performed using units of noise exposure as a continuous regressor, and age as a regressor of no interest, to explain the variance in sound-related activity.

A secondary objective is to investigate whether there are any additive contributions to these physiological changes attributable to tinnitus or diminished sound-level tolerance, conditions which are often comorbid with hearing problems. The linear regression model will be expanded to a stepwise multiple regression modeling to examine the relative additional contributions of tinnitus and reduced sound-level tolerance to the total variance explained.

#### Missing Data

Any participants that have contraindications for MRI will be excluded as stated in the protocol. Analyses will be based on all observed data, but the study team will be particularly vigilant to reduce missing data. The number of these participants excluded, and those with missing data, will be reported in subsequent publications.

#### Incidental Findings

As the individuals this study aims to recruit are healthy, it is extremely unlikely that any MRI scan will show an abnormality. Furthermore, MRI scans will not be routinely inspected by a neuroradiologist. However, if a researcher working on the study did suspect that there was something abnormal on a scan, then the images will be sent to a neuroradiologist who will contact the participant’s GP if they decide that the scan needs further investigation.

Likewise, as the study aims to recruit individuals with normal hearing, it is not anticipated that any hearing losses measured will be severe enough to warrant concern or further investigation. If any individual is concerned by the outcome of investigation by audiometry, tympanometry, or otoscopy, the participant will be recommended that they should see their GP or visit an audiologist.

## Results

The MRC-funded program was awarded in July 2013. Enrollment for the study described in this protocol commenced in February 2017 and was completed in December 2017. Results are expected in 2018.

## Discussion

### Dissemination Plan

To ensure maximum reach of results throughout the clinical and research communities, all work will be presented at the annual conferences of the British Society of Audiology, the Association for Research in Otolaryngology, the Tinnitus Research Initiative, and the International Society of Magnetic Resonance in Medicine, and published in peer-reviewed otolaryngology and neuroimaging journals, with Open Access. Additionally, plain language descriptions of the key findings and clinical implications will be summarized in newsletters and social media channels published by patient-facing organizations such as American Tinnitus Association, Action on Hearing Loss, British Tinnitus Association, and TinnitusHub. Anonymized raw or processed data can be made available to interested parties through communication with the corresponding author.

### Conclusions

The imaging study described in this protocol seeks to provide the first comprehensive characterization of the physiological effects of noise exposure on the brains of audiometrically normal humans within major structures of the ascending auditory pathway. Our findings have the potential to inform diagnosis and prevention of hearing problems due to noise exposure. In this final section, we speculate on what those future gains might be. With respect to diagnosis, the results of this study have the potential to lead to patient benefit through early identification of cochlear damage not yet measurable by pure tone audiometry. Depending on our findings, it may be that in the future, such MRI and ABR procedures should always be used in conjunction with other available objective clinical diagnostics, such as otoacoustic emission testing, which can be important for determining subclinical dysfunctions at the level of the outer hair cells, efferent feedback control system, and the olivocochlear nucleus [18]. As such, individuals presenting with symptoms characteristic of hidden hearing loss or early signs of noise-induced cochlear synaptopathy may be offered a more informative investigation with the potential of a more specific diagnosis. With respect to prevention, identification of at-risk individuals through early detection will enable improved and personalized health care advice, promoting behaviors that improve long-term hearing health, such as increased use of ear protection. Additionally, evidence from this research can be used to determine exposure levels that are safe for the majority of individuals. This may lead to an alteration (lowering) of the current occupational noise exposure guidelines or regulations, and increased monitoring of individuals who approach unsafe exposure levels, with the advantage of greater diagnostic power afforded by the techniques outlined in this report. These latter two mechanisms in turn will lead to prevention of noise-induced hearing loss, thereby reducing the demands on health care resources.

## Abbreviations

ABR: auditory brainstem response
AEP: auditory evoked potential
BOLD: Blood-Oxygen-Level Dependent
CN: cochlear nucleus
CSF: cerebrospinal fluid
EEG: electroencephalography
EPI: echo-planar imaging
FLASH: fast low angle shot
fMRI: functional magnetic resonance imaging
GE: gradient echo
IC: inferior colliculus
MGB: medial geniculate body
MNI: Montreal Neurological Institute
MPRAGE: magnetization prepared rapid acquisition gradient echo
MRI: magnetic resonance imaging
SENSE: sensitivity encoding
SPIRIT: Standard Protocol Items: Recommendations for Interventional Trials
(SPMIC: Sir Peter Mansfield Imaging Centre
SPL: sound pressure level
TE: echo time
TR: repetition time
TSE: turbo spin echo
